# Recombinant Lectin from Tepary Bean (*Phaseolus acutifolius*) with Specific Recognition for Cancer-Associated Glycans: Production, Structural Characterization, and Target Identification

**DOI:** 10.3390/biom10040654

**Published:** 2020-04-23

**Authors:** Dania Martínez-Alarcón, Annabelle Varrot, Elaine Fitches, John A. Gatehouse, Min Cao, Prashant Pyati, Alejandro Blanco-Labra, Teresa Garcia-Gasca

**Affiliations:** 1 Centro de Investigación y de Estudios Avanzados Unidad Irapuato, Departamento de Biotecnología y Bioquímica, Irapuato 36821, Guanaj uato, Mexico; dania.martinez.alarcon@gmail.com; 2 University of Grenoble Alpes, CNRS, CERMAV, 38000 Grenoble, France; annabelle.varrot@cermav.cnrs.fr; 3 Department of Biosciences, Durham University, Durham DH1 3LE, UK; e.c.fitches@durham.ac.uk (E.F.); j.a.gatehouse@durham.ac.uk (J.A.G.); mincao.chn@gmail.com (M.C.); prashpya@googlemail.com (P.P.); 4 Facultad de Ciencias Naturales, Universidad Autónoma de Querétaro, Santiago de Querétaro 76230, Querétaro, Mexico

**Keywords:** recombinant lectins, Tepary bean, *Pichia pastoris*, MGAT5, glycan array, structure, cancer

## Abstract

Herein, we report the production of a recombinant Tepary bean lectin (*r*TBL-1), its three-dimensional (3D) structure, and its differential recognition for cancer-type glycoconjugates. *r*TBL-1 was expressed in *Pichia pastoris,* yielding 316 mg per liter of culture, and was purified by nickel affinity chromatography. Characterization of the protein showed that *r*TBL-1 is a stable 120 kDa homo-tetramer folded as a canonical leguminous lectin with two divalent cations (Ca^2+^ and Mn^2+^) attached to each subunit, confirmed in its 3D structure solved by X-ray diffraction at 1.9 Å resolution. Monomers also presented a ~2.5 kDa *N*-linked glycan located on the opposite face of the binding pocket. It does not participate in carbohydrate recognition but contributes to the stabilization of the interfaces between protomers. Screening for potential *r*TBL-1 targets by glycan array identified 14 positive binders, all of which correspond to β1-6 branched *N*-glycans’ characteristics of cancer cells. The presence of α1-6 core fucose, also tumor-associated, improved carbohydrate recognition. *r*TBL-1 affinity for a broad spectrum of mono- and disaccharides was evaluated by isothermal titration calorimetry (ITC); however, no interaction was detected, corroborating that carbohydrate recognition is highly specific and requires larger ligands for binding. This would explain the differential recognition between healthy and cancer cells by Tepary bean lectins.

## 1. Introduction

Lectins are proteins that bind to carbohydrates, either free or bound to cell membranes as part of glycoproteins, glycolipids, or polysaccharides. Since these interactions are reversible and highly specific, lectins have been widely used to elucidate alterations in the composition of carbohydrates during pathological processes such as cancer, where the cell glycosylation machinery is frequently altered, leading to the exposure of aberrant carbohydrates on cellular surfaces [1,2,3,4,5].

Given that cancer is a microevolutionary process, anomalous carbohydrate edition does not occur randomly; in fact, there is a limited subset of changes that correlate with malignant transformation [5]. These changes vary depending on the cancer type and are often related to tumor progression and lifespan [4]. Some of the most common tumor-associated glycan changes described to date include (1) increased *β*1-4 branched tetra-antennary *N*-glycans [4,5]; (2) *α*2-6 sialylation on the outer poly-*N*-acetyllactosamines (LacNAc) of *N*-glycans [6]; (3) increase of *α*1-6 fucosylation [7,8]; (4) overexpression of mucins and truncated *O*-glycans [9]; (5) altered expression of blood groups and sialyl-Lewis antigens [10]; (6) increase in bisecting GlcNAc [11]; (7) overexpression of hyaluronan [12,13]; and (8) increased β1-6 branching of *N*-glycans [4,5]. The latter is one of the most important glycan modifications in colorectal cancer cells. It is derived from an upregulation of the N-acetylglucosaminyltransferase V (MGAT5) by oncogenic transcription factors from the RAS–RAF–MAPK signaling pathways [4], which are highly associated with cancer metastasis [11].

A possible mechanism by which MGAT5 upregulation enhances cancer progression is by causing a high frequency of branched *N*-glycans on the extracellular domains of Receptor Tyrosine Kinases (RTKs) [14,15]. RTKs are the second main type of cell surface receptors, and they are critical actors in cancer through regulation of multiple cellular processes such as growth, migration, proliferation, differentiation, and apoptosis [15]. Activation of these receptors occurs through cross-linked phosphorylation after oligomerization induced by ligand binding. Their action is regulated by a fine balance between their synthesis/exposure and internalization/degradation. Alterations in the glycosylation pattern of RTKs lead to the disruption of this balance, allowing, for example, longer retention on the surface of cancer cells. Such is the case of the epidermal growth factor receptor (EGFR), of which recognition by galectins promotes lattice formation, enhances phosphorylation, and causes a high rate of cell proliferation via signal transduction [14,16]. Consequently, targeting RTK recognition represents an interesting therapeutic strategy to block cell growth and may provide new strategies for unique and combinatorial therapies.

It has been shown that a semi-pure fraction of lectins from Tepary bean (TBLF), mainly composed of two different glycoproteins (TBL-1 and TBL-2), can recognize a broad spectrum of human cancer cells. It induces apoptosis at low concentrations; the most sensitive are colon and breast cancer cells. TBLF lectins are highly specific in distinguishing between normal and cancer cells; thus, as a consequence, a differential cytotoxic effect is displayed [17] by apoptosis induction and cell cycle arrest [18]. These lectins are particularly resistant and can cross the gastrointestinal tract without being degraded by pH conditions and/or digestive enzymes [19]. Furthermore, TBLF presents low toxicity and inhibits early tumorigenesis in rats with chemically induced colorectal cancer [19,20]. When administered intragastrically to rats, TBLF has been shown to have a stimulatory effect on the immune system and displays few side effects, such as atrophy in small intestine villus and colonic crypts and pancreatic hypertrophy, that can be partially reverted after a two-week rest period [21]. These results suggest the potential therapeutic use of Tepary bean lectins against colon cancer.

The main drawback of the use of TBLF as an anticancer therapy is that its purification from seeds is slow and expensive and provides low yields. Its purification comprises a six-step train, where large amounts of methanol, chloroform, and ammonium sulphate are needed, among others. One of the major limitations of this process is that the gel filtration columns, which are approximately 2 m long, are run by gravity and that only a small amount of the sample can be processed at a time, resulting in a very time-consuming step. Our general estimates indicate that obtaining 1 g of semi-pure TBLF requires 30–41 weeks of work for one person, 1 kg of beans, 15 L of chloroform, 5 L of methanol, and 1.7 kg of ammonium sulphate, among other reagents on a smaller scale. When a high-purity sample is required, it is necessary to perform an additional step of purification by ion exchange chromatography and HPLC, which further reduces the performance.

TBLF lectins are complex glycoproteins, and preliminary data suggest that posttranslational modifications could influence folding and, consequently, could alter their biological function. Previously, we reported the cisgenic expression of a TBLF lectin using a strategy that allowed its secretion through root exudates of genetically modified Tepary bean plants [22]. However, complete plant regeneration is a complex process and the yield obtained using this approach (~21 µg of protein per gram of dry root) was insufficient to meet the protein requirements for in vivo assays, requiring investigation of alternative means of production.

Since bacteria lack organelles for posttranslational processing, yeasts are the most common system for the expression of glycoproteins, such as legume lectins. Initially, the most promising system for this purpose was *Saccharomyces cerevisiae* due the knowledge of its genetic manipulation [23,24]. However, *S. cerevisiae* has the disadvantage of producing high mannose *N*-glycans with outer chains of typically 50–150 residues in length (~>9 to ~27 kDa), which are highly antigenic for humans [23,25]. Thus, the methylotrophic yeast *Pichia pastoris,* which does not appear to add extra α1-3 mannose residues or to carry out hyperglycoyslation on its *N*-glycans, has been successfully used as expression system for leguminous lectins [26,27]. In addition, recombinant proteins produced by *Pichia* can be directly recovered from the culture media by coupling them to the *S. cerevisiae* α-factor, which is a signal peptide that redirects the protein to the secretory pathway and is subsequently cleaved in the Golgi by *Pichia* endopeptidases [24].

In this work, we present the production of a recombinant lectin from Tepary bean (rTBL-1) by using *P. pastoris* engineering, its biochemical characterization, crystallographic structure, and an analysis of its specificity for cancer-type glycans.

## 2. Materials and Methods

### 2.1. Materials

Chemicals and reagents were of analytical grade and were supplied by Sigma (Sigma-Aldrich, Gillingham, UK) or BDH Chemical Company, unless stated otherwise. Restriction enzymes were supplied by Fermentas (Ontario, Canada) and antibodies from Invitrogen, Thermo Fisher Scientific (Carlsbad, CA, USA), and Bio-Rad Laboratories (Hercules, CA, USA).

### 2.2. Strains

Gene constructs were prepared in the TOP10 strain of *Escherichia coli*, and the production of proteins was carried out using the *P. pastoris* protease-deficient strain SMD1168H.

### 2.3. Plasmids

pGAPZαB plasmid was provided by Invitrogen Life Technologies (Carlsbad, CA, USA). Briefly, this commercial plasmid allows the transformation of *P. pastoris* by recombining the polylinker flanking sequences. Expression of the cloned genes is under regulation of the constitutive Glyceraldehyde-3-phosphate dehydrogenase (GAP) promoter. This vector also contains replication sites for both *E. coli* and *P. pastoris*, a zeocin (Zn) resistance marker, and a *S. cerevisiae* signal peptide for protein secretion (α-factor) fused to the *N*-terminal of the protein and subsequently cleaved by endopeptidases Kex2 and Ste13 in the Golgi apparatus.

### 2.4. Tepary Bean Lectin Fraction (TBLF)

TBLF was obtained by purification from Tepary bean seeds. Briefly, beans were finely ground and the resulting flour was defatted by several washes with a mixture of CHCl_3_/MeOH (3:1) until the filtering was clear. Then, total proteins were extracted by using Tris-HCl pH 8.0 at 4 °C, and a sequential precipitation was performed with ammonium sulphate at 40% and 60% (*w*/*v*) saturation, followed by intensive dialysis. Finally, the protein extract was separated by molecular size exclusion chromatography using a G-75 Sephadex column [17,18].

### 2.5. Vector Construction

The coding sequence of *r*TBL-1 was amplified with primers (forward 5′-TATCTGCAGCATCAGCCAACGACATCTC-3′ and reverse 5′-ATATCTAGACTA ATGATGATGATGATGATGATGATTC-3′), where PstI and XbaI restriction sites to the 5′ and 3′ end of the sequence were added (underlined sequences). Next, both the amplicon and pGAPZαB vector were digested with PstI and XbaI enzymes for 3 h at 37 °C and then purified from gel electrophoresis using the commercial kit of BioLabs (Hitchin, UK) “Monarch DNA Gel Extraction.” The products obtained were ligated using T4 ligase in a 3:1 insert/vector ratio. The reaction was incubated at 25 °C for 1 h, and the product was used to transform the electrocompetent cells of *E. coli* TOP10 using an electroporator with a resistance of 100 Ohms, capacitance of 125 μFa, and 1.8 Volts. The transformed cells were recovered for growth on plates of Luria–Bertani (LB) medium with 25 μg/mL zeocin as a selection agent and incubated at 37 °C for 24 h. The transformants were screened by colony PCR using a set of primers designed to hybridize the flanking sequences of the pGAPZαB polylinker (pGAP Forward 5′-GTCCCTATTTCAATCAATTGAA-3′ and AOX1 5′-GCAAATGGCATTCTGACATCC3-3′). Plasmid extraction was carried out from one of the positive colonies, and the in-frame insertion and the correct coding sequence were verified by digestion and DNA sequencing.

### 2.6. Transformation of Pichia pastoris

Once the sequence was confirmed, the plasmid was digested with AvrII enzyme at 37 °C for 16 h and complete DNA linearization was confirmed by nucleic acid electrophoresis. Subsequently, the DNA was precipitated (100% ethanol and 5 M ammonium acetate at −20 °C for 16 h), centrifuged, and resuspended in 20 μL of nuclease-free sterile water, and then, a concentration >5 μg/μL was confirmed by NanoDrop spectrophotometry (NanoDrop 2000c from Thermo Fisher Scientific; Hercules, CA, USA) and electrophoresis. This material was used to transform *P. pastoris* by means of the Pichia EasyCompTM Transformation Kit (Thermo Fisher Scientific; Pittsburgh, PA, USA), according to the manufacturer’s protocol. Transformed cells were recovered for growth on plates of Yeast Extract–Peptone–Glycerol (YPG) medium with 50 μg/mL of zeocin and were incubated at 30 °C for 48 h.

### 2.7. Production

Ten of the transformed *P. pastoris* colonies were inoculated in 10 mL of liquid YPG medium containing 25 µg/mL of zeocin. Cultures were allowed to grow at 30 °C with shaking for 48 h, and then, the supernatants were recovered by centrifugation at 11,800× *g* for 10 min. Aliquots of 25 µL of each of the supernatants were used for SDS-PAGE electrophoresis [28], followed by semi-dry transfer (ATTO blotter, Tokyo, Japan) for 1 h at constant voltage (10 V) to nitrocellulose membranes previously soaked in 1X TBS. The membranes were stained with Ponceau red to indicate the position of the molecular weight markers and then subsequently immersed in blocking solution (defatted milk powder 5% *w*/*v* in 0.1% *v*/*v* Tween TBS) for 1 h. After blocking, the membranes were incubated overnight with a 6XHis Tag mouse monoclonal antibody (Invitrogen, Carlsbad, CA, USA) in a 1:1000 dilution prepared in blocking solution. After 16 h, the primary antibodies were removed by several washes with blocking solution, and then, the membranes were incubated for 2 h in blocking solution containing HRP-conjugated secondary goat anti-mouse antibodies (Bio-Rad, Hercules, CA, USA). Finally, immunoreactivity was visualized by chemiluminescence.

### 2.8. Heterologus Expression and Purification of rTBL-1

For protein production, *P. pastoris* (SMD1168H) cells expressing recombinant *r*TBL-1 were grown in an Applikon ez-control laboratory fermenter (7.5 L vessel) as described previously [29], except that the pH was maintained at 5.0. The secreted proteins were separated from cells by centrifugation (30 min, 7500× *g*, 4 °C), and the supernatant was clarified by sequential filtering with 2.7 and 0.7 μM glass fiber filters (Whatmann, Maidstone, UK). Recombinant rTBL-1 was purified from the supernatant by nickel-affinity chromatography by using HisTrap HP columns (GE Healthcare, Maidstone, UK) dialysed and freeze-dried as described [30]. The protein contents in lyophilized samples were determined from SDS-PAGE gels based on bands corresponding to intact proteins, which were compared to GNA (Sigma) standards by visual inspection, and by capturing an image of the de-stained gel using a commercial flat-bed scanner; image analysis was carried out with a custom-written software program (ProQuantify version 2.0 supplied by Rodrigo Guerrero).

### 2.9. Molecular Size Exclusion Chromatography

Molecular size exclusion chromatography (SEC) was performed using a High-Resolution ENrich™ SEC 650 column (Bio-Rad, Marnes-la-Coquette, France) on the NGC™ chromatography system (Bio-Rad, Marnes-la-Coquette, France). Prior to assay, the column was calibrated using the gel filtration standard #15119001 (Bio-Rad, Marnes-la-Coquette, France), according to the supplier’s instructions, and protein samples at 10 mg/mL were centrifuged for 30 min at 12,000 rpm. The column was equilibrated with 50 mL of buffer D (20 mM (2-(*N*-morpholino)ethanesulfonic acid, MES) pH 6.5 and 100 mM NaCl), and 200 µL of the sample was injected into the system, followed by 30 mL isocratic elution on buffer D. The fractions were monitored by absorbance at 280 nm and collected every 0.5 mL.

### 2.10. Thermal Shift Assay (TSA)

The thermal stability of rTBL-1 was analyzed by TSA using a MiniOpticon real-time PCR system (Bio-Rad, Marnes-la-Coquette, France). Prior to assay, buffer stocks at 100 mM and a mixture containing 70 μL of rTBL-1 at 1 mg/mL, 7 μL of 500X Sypro Orange (Sigma-Aldrich, Saint Quentin Fallavier, France), and 63 μL of H_2_O were prepared. Then, 7.5 μL of H_2_O, 12.5 μL of the corresponding buffer and 5 μL of the protein/Sypro mixture were mixed in 96-well PCR microplates. The heat exchange test was then carried out from 20 to 100 °C with a heating rate of 1 °C/min. Fluorescence intensity was measured with Ex/Em: 490/530 nm, and data processing was performed using CFX Manager software version 1.6.

### 2.11. Determination of the Polypeptidic Sequence

The product of the translation as well as the most frequent *C*- and *N*-terminal residues were determined by nano-LC–MS/MS, depending on standard data of tryptic and chemotryptic peptides generated from the digestion of the samples, where a method of acquisition of data-dependent mass spectrometers was used. Along a reverse phase gradient used for the separation of peptides, the MS scans were followed using an MS/MS spectrum from the selected precursors. The mobile exclusion windows prevented the reacquisition of the same precursors during a fixed period and allowed to collect less-abundant ion data. For detection of glycosylated peptides, the same methodology was followed on tryptic peptides of deglycosylated protein.

### 2.12. Identification of the Glycosylations Present in the Recombinant Lectin

Proteins were subjected to glycan digestion using combinations of five different specific glycosidases for *O-* and *N*-glycosylations using the Glycoprotein Deglycosylation kit (EMD Millipore, Danvers, MA, USA). The antennas’ size was determined through the retention factor (RF) of samples on SDS-PAGE, both before and after deglycosylation. To validate these results, the periodic acid-Schiff reagent (PAS) staining technique was used (Sigma Chemical, St. Louis, MO, USA), as described by the supplier. The glycosidases were (1) *N*-Glycosidase F: this enzyme cleaves all *N*-linked oligosaccharides, unless their core is α1-3 fucosylated; (2) Endo-α-*N*-acetylgalactosaminidase that separates common nucleus (Galβ1-3GalNAca) from all *O*-glycosylations (it only works when no additional sugars adhere to the main nucleus); (3) α2-3,6,8,9-neuraminidase that cuts all sialic acids that may be attached to the common core of the *O*-glycosylations; (4) β1-4galactosidase that releases only non-reducing terminal galactose, bound in β1-4 to *O*-glycosylations; and (5) β-*N*-acetylglucosaminidase that cleaves all GlcNAc residues bound to β-non-reducing terminal *O*-glycosylations.

### 2.13. Crystallization and Data Collection

Crystal screening was performed using the hanging-drop vapor diffusion technique by mixing equal volumes of pure protein at 5 mg/mL and precipitant solutions from the BCS Screen (Molecular Dimensions, Newmarket, Suffolk, UK). Drops were incubated at 19 °C until crystals appeared. A subsequent optimization of positive conditions was carried out, and crystals suitable for X-ray diffraction analysis were obtained under a solution containing 100 Mm Tris-HCl buffer pH 7.5 and 18% (*v*/*v*) PEG Smear Low (mix containing PEG 400, PEG 550 MME, PEG 600, and PEG 1000). The crystals were soaked in mother liquor supplemented with 30% (*v*/*v*) of PEG Smear Low (Molecular Dimensions, Newmarket, Suffolk, UK) prior to flash cooling in liquid nitrogen. Data collection was performed on the beamline Proxima-1 at SOLEIL Synchrotron, Saint Aubin, France using an Eiger X 16M detector.

### 2.14. Structure Determination

Diffraction data were processed using XDS [31] and converted to structure factors using the CCP4 program package v.6.1 [32] with 5% of the data reserved for R_free_ calculation. The structure of the *r*TBL-1 was solved by molecular replacement using Phaser MR v.2.5 [33], utilizing the tetramer coordinates of PHA:L (PDB entry 1 FAT A) [34] as the search model after trimming with Chainsaw. Restrain refinement was performed using REFMAC5 [35] alternated with manual model building in Coot v.0.7 [36]. The sugar residues and other compounds that were present were placed manually using Coot. Water molecules were added automatically in Coot and checked manually. The final structure was validated using the validation server from the Protein Data Bank (PDB) (https://validate-rcsb-1.wwpdb.org/) and was deposited in the PDB as entry 6TT9.

### 2.15. Glycan Array

*r*TBL-1 was labeled with fluorescein isothiocyanate (FITC) (Sigma-Aldrich, St Louis, MO) according to the supplier’s instructions with slight modifications. Briefly, 2 mg of protein was dissolved in 1 mL of buffer E (100 mM Na_2_CO_3_ and 100 mM NaCl, pH 9); then, 40 μL of FITC at 1 mg/mL in dimethyl sulfoxide (DMSO) was gradually added to the protein solution, and the mixture was gently stirred at room temperature overnight. The next day, the solution was supplemented with NH_4_Cl to a final concentration of 50 mM and free FITC was removed using a PD10 desalting column (GE Healthcare Life Sciences) with PBS as the mobile phase. The protein concentration was determined at ABS_280_ and FITC at ABS_490_ using a NanoDrop^TM^ 200 (Thermo Fisher Scientific), and the Fluorescein/Protein molar ratio (F/P) was estimated by the following formula:

$$\mathrm{Molar}\frac{F}{P}=\frac{\mathrm{MW}}{389}\times \frac{\frac{A_{495}}{195}}{\left[A_{280}-\left(0.35\times A_{495}\right)\right]{\;E}^{0.1\%}}$$

where MW is the protein molecular weight, 389 is the FITC molecular weight, 195 is the absorption E0.1% of bound FITC at 490 nm and pH 13.0, (0.35 × A_495_) is the correction factor due to the absorbance of FITC at 280 nm, and E0.1% is the absorption at 280 nm of a protein at 1.0 mg/mL (an ideal F/P should be 0.3 > 1).

Labeled *r*TBL-1 was sent to the Consortium for Functional Glycomics (CFG) (Boston, MA, USA), and the binding properties were assayed at 5 and 50 μg/mL on a “Mammalian Glycan Array version 5.4” that contain 585 glycans in replicates of six. The highest and lowest signals of each set of replicates were eliminated, and the average of the remaining data was normalized to the percentages of the highest relative fluorescent units (RFU) value for each analysis; finally, the percentages for each glycan were averaged at different lectin concentrations.

### 2.16. Isothermal Titration Calorimetry (ITC)

Experiments were performed using a Microcal ITC200 calorimeter (Malvern Panalytical, Malvern, UK) with 40 μL of ligand and 200 μL of rTBL-1 100 µM. Both the protein and the ligand were dissolved in MES buffer pH 6.5, and the sugar was gradually added to the sample cell by 2 µL injections in a range of 120 s while stirring at 1000 rpm. The experimental data were adjusted to a theoretical titration curve by the Origin ITC analysis software.

## 3. Results

### 3.1. Production of Recombinant TBL1 (rTBL-1)

**Figure 1:**
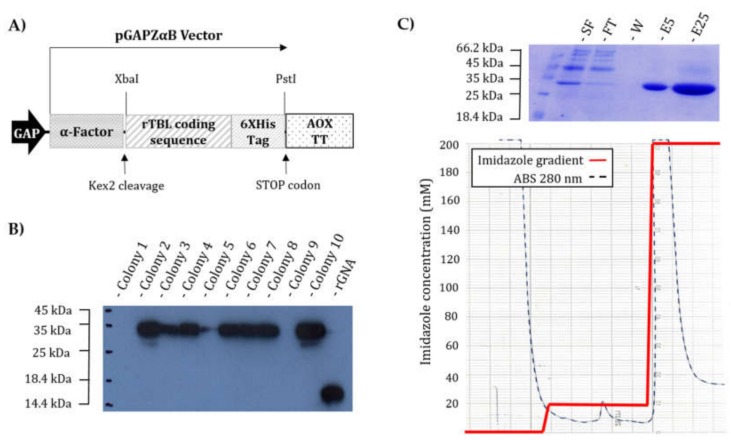
*r*TBL-1 production and purification: (**A**) Schematic representation of the construct encoding for *r*TBL-1 in the vector pGAPαB-*r*TBL-1. The α-factor pre-pro sequence directs the expressed protein to the yeast secretory pathway, enabling purification from fermented culture supernatants. (**B**) Western blot screening with anti-His antibodies to identify yeast clones expressing *r*TBL-1: lane 1, ladder; lanes 2–11, transformed colonies; and lane 12, 500 ng of recombinant histidine-tagged GNA as a positive control. (**C**) *r*TBL-1 purification process: top: SDS-PAGE electrophoretic profiles with gel stained for total protein. Lane 1, ladder; lane 2, 25 μL of culture supernatant; lane 3, 25 μL of flow through; lane 4, 25 μL of the wash with 10 mM imidazole buffer; and lanes 5 and 6, 5 and 25 μL of elution with 200 mM imidazole buffer, respectively. Bottom: chromatogram with imidazole gradient and Abs_280_ represented with red and black dotted lines, respectively.

A synthetic gene encoding for the mature sequence of TBL-1 was inserted into the pGAPZαB expression vector under regulation of the constitutive GAP promoter by merging its *N*-terminus to the yeast α-factor sequence using XbaI and PstI restriction enzymes (Figure 1A). Plasmid DNA from a sequence-verified clone was then linearized and the expression cassette integrated at the *GAPDH* locus of *P. pastoris* by DNA recombination. Western blotting of shake flask culture supernatants showed that eight of the ten selected colonies expressed and secreted the lectin, with at least seven colonies displaying relatively high expression levels (Figure 1B). The expression under shake flask conditions was estimated to be 2–32 μg/mL, with colonies 5 and 10 being the lowest and highest producers, respectively. Production was then scaled in a 7-L bench top fermenter, where a final yield of 316 ± 0.27 mg recombinant lectin/L of culture supernatant was obtained using colony 10 as the pre-culture. *r*TBL-1 was purified by nickel affinity chromatography, and fractions were collected after elution with 200 mM imidazole. The analysis of purified *r*TBL-1 by SDS-PAGE electrophoresis showed the presence of a single band of ~30 kDa in gels stained for total proteins (Figure 1C). No additional protein bands or degradation products were identified, even when the wells were loaded with ~50 μg of protein.

### 3.2. rTBL-1 Characterization

**Figure 2:**
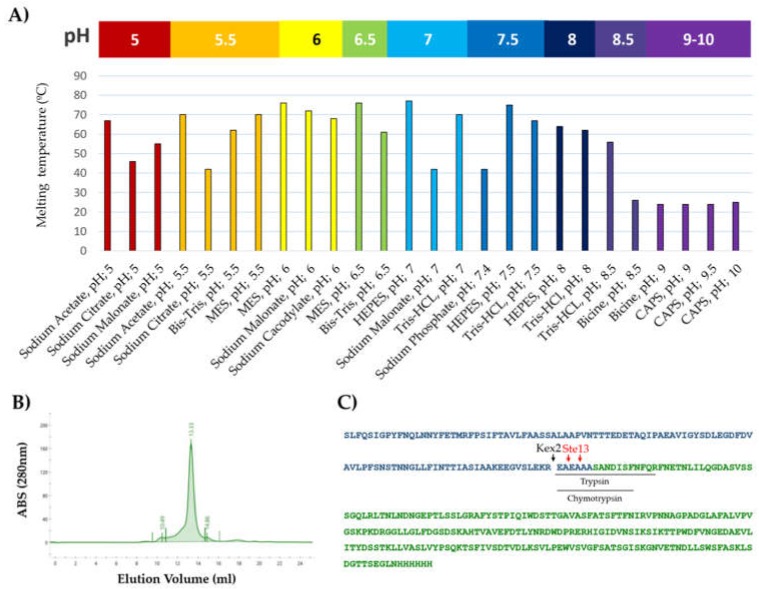
Characterization of *r*TBL-1: (**A**) Melting temperatures of *r*TBL-1 obtained through thermal shift assay (TSA). A temperature gradient from 20 to 100 °C was applied under 26 different buffer conditions in a pH range from 5 to 10. (**B**) Size exclusion chromatogram of rTBL-1: The column was calibrated using the gel filtration standard #15119001 (Bio-Rad). (**C**) Sequence of *r*TBL-1 by mass spectrometry; green depicts *r*TBL-1 sequence; blue represents α-factor sequence; black lines indicate the most abundant tryptic and chemotryptic N-terminal peptides found in the digested samples; and cleavage sites for Kex2 and Ste13 are indicated by black and red arrows, respectively.

A thermal shift assay (TSA) was used to evaluate the stability of the protein through a temperature gradient from 20 to 100 °C using 26 different buffers in the pH range of 5–10. The most stable conditions were obtained with MES, Tris, and HEPES buffers in a pH range of 6–7.5, with melting temperatures of 76, 70, and 75 °C, respectively. Citrate, phosphate, and malonate buffers in the same pH range displayed melting temperatures <45 °C. Interestingly, *r*TBL-1 was stable up to 55 °C at pH 8.5 in Tris-HCl buffer, whereas it was denatured at a temperature <25 °C in 3-(Cyclohexylamino)-1-propanesulfonic acid (CAPS) and Bicine buffers at the same pH (Figure 2A).

To evaluate if the heterologous expression of *r*TBL-1 could affect its ability to form tetramers, its molecular weight was determined by gel filtration using a high-resolution size exclusion column, ENrich™ SEC 650 (Bio-Rad). The elution profile shows a single peak corresponding to a protein of ~120 kDa and indicating that the protein formed a homo-tetramer in the solution (Figure 2B).

The *r*TBL-1 sequence and α-factor excision were verified by mass spectrometry in two samples independently digested with trypsin and chymotrypsin. The MS/MS spectra acquired after trypsin digestion matched the expected sequence of the protein. Regarding the α-factor, different peptides overlapping the Kex2p and Ste13p cleavage sites were observed, but by far, the most abundant spectra was for the *N*-terminal peptide EAEAAASANDISFNFQR (Figure 2C). This peptide can be generated by trypsinolysis itself or by combined trypsin and Kex2p activity, since Kex2p also cleaves at the *C*-terminal of arginine residues. Nevertheless, the prevalence of the spectrum matches the peptide EAEAAASANDISF, produced by chymotrypsin digestion, confirming the *N*-terminal sequence of the protein. The peptide AASANDISF, derived from Ste13p cleavage, was also identified in both samples; however, its small proportion indicates that most of the protein was cleaved exclusively by the Kex2p enzyme.

### 3.3. Comparison of rTBL-1 with Native TBL-1

**Figure 3:**
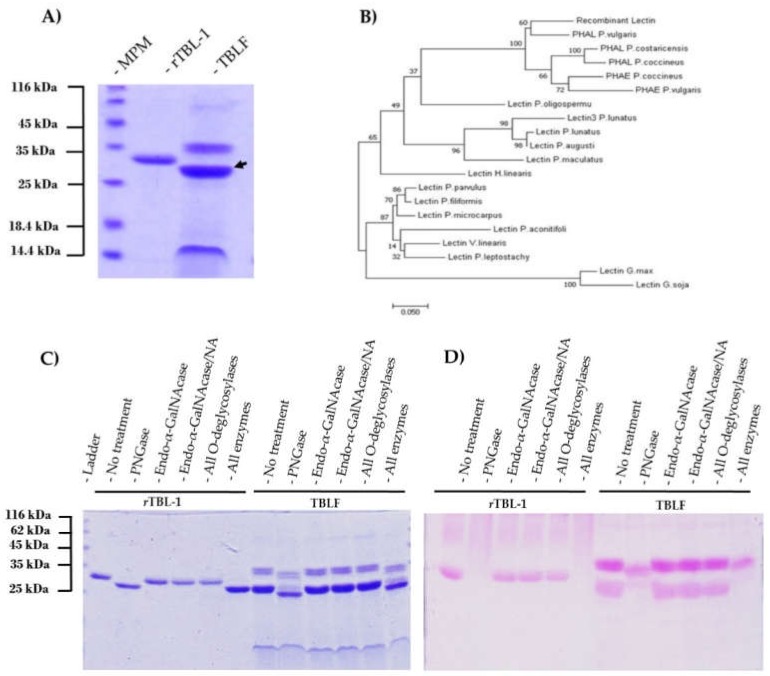
Molecular comparison of recombinant and native TBL-1: (**A**) SDS-PAGE electrophoresis of *r*TBL-1 and Tepary Bean Lectin Fraction (TBLF) (gels stained for total protein). The black arrow shows native TBL-1. (**B**) Comparative phylogenetic sequence analysis of several legume lectins. (**C**) SDS-PAGE of *r*TBL-1 and Tepary bean lectin fraction (TBLF) +/− deglycosylation treatments. Lane 1, *r*TBL-1 no enzyme control; lanes 2–5, *r*TBL-1 treated with PNGaseF, endo-α-GalNAcase, endo-α-GalNAcase, and neuraminidase (NA), respectively; lane 6, *r*TBL-1 treated with all *O*-deglycosylases (β1,4-galactosidase, endo-α-GalNAcase, NA, and β-N-acetylglucosaminidase); lane 7, *r*TBL-1 treated with all deglycosylases (*N*-glycosidase F, β1,4-galactosidase, endo-α-GalNAcase, NA, and β-*N*-acetylglucosaminidase); lane 8, TBLF without treatment; lanes 9–11 TBLF treated with PNGaseF, endo-α-GalNAcase, endo-α-GalNAcase, and NA, respectively; lane 12, TBLF treated with all O-desglycosylases (β1,4-galactosidase, endo-α-GlcNAcase, NA, and β-*N*-acetylglucosaminidase); lane 13, TBLF treated with all deglycosylases (*N*-glycosidase-F, β1,4-galactosidase, endo-α-GalNAcase, NA, and β-*N*-Acetylglucosaminidase). (**D**) SDS-PAGE of *r*TBL-1 and TBLF +/– deglycosylation treatments stained with Schiff-PAS reagent for the detection of glycoproteins. Sample arrangement is as described for SDS-PAGE gel.

According to the electrophoretic profile, *r*TBL-1 is ~2.5 kDa bigger than native TBL-1 (Figure 3A). This difference in size is mainly attributed to three factors: (1) the addition of a 6XHis tag (~840.92 Da); (2) the sequence EAEAAA at the *N*-terminal, derived from Kex2p cleavage of the α-factor (~561 Da); and (3) the presence of glycosidic antennas ~640 Da bigger than those on TBL-1.

A comparative sequence analysis of several legume lectins with high homology to TBL-1 was performed (Figure 3B). The taxa inferred by the evolutionary analysis showed that TBL-1 belongs to the group of phytohemagglutinins of the *Phaseolus* genus; more specifically, the data suggest that TBL-1 is actually a homologue of PHA:L, a leucoagglutinin from *Phaseolus vulgaris*, present in four of the five different populations of PHA heterotetramers (E4, E3L, E2L2, EL3, and L4) [37,38].

It is important to highlight that, while TBL-1 is a protein that binds carbohydrates, it is also a glycoprotein that contains covalently attached glycans. The structure and size of these carbohydrates play an important role in protein folding and, consequently, a gross alteration of its pattern during heterologous expression into *Pichia pastoris* could disrupt activity. Therefore, glycan characterization via treatment of *r*TBL-1 and TBLF with glycosidases, followed by SDS-PAGE and Schiff-PASS staining, was conducted (Figure 3C,D). When treated with *N*-glycosidase-F, a size reduction of ~2.48 and ~1.84 kDa for rTBL-1 and TBL-1, respectively, was observed, accompanied by the complete loss of their carbohydrate moiety. No loss of intensity or size was detected after treatment with *O*-glycosidases in any sample, suggesting that both proteins contain exclusively *N*-linked glycans. To validate the absence of *O*-linked glycans and to confirm the position of the carbohydrate antennas of *r*TBL-1, LC–MS/MS was performed after trypsinolysis of samples +/− deglycosylation. Peptides with confidence scores >95% in both samples were compared to identify those present only after deglycosylation, and it was found that 63.45% corresponded to peptides containing the *19*-NETN-*23* sequence. It is noteworthy that almost all of the present peptides showed deamidation at Asn19, known to be a product of *N*-glycosidase-F treatment.

### 3.4. Structure Determination

**Figure 4:**
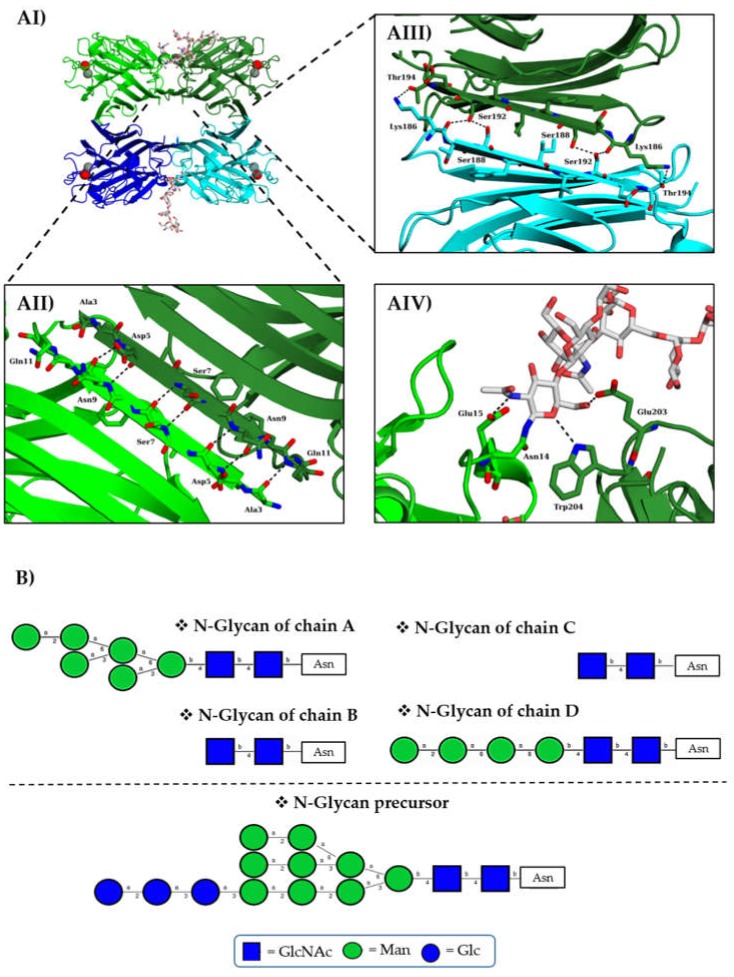
Structure, N-glycosylation, and interface interactions in *r*TBL-1 tetramer: (**AI**) rTBL-1 tetramer colored by chains with chains A, B, C, and D in light green, dark green, dark blue, and light blue, respectively, with Ca^2+^ depicted in red spheres and Mn^2+^ depicted in grey spheres. (**AII**) Stabilizing interactions of the interface between A and B chains. (**AIII**) Stabilizing interactions of the interface between B and D chains. (**AIV**) GlcNAc interactions in the interface of two adjacent chains. (**B**) Structure of the *N*-glycans identified in each monomer of *r*TBL-1.

*r*TBL-1 was crystallized and its structure was solved by molecular replacement at 1.9 Å using the PHA:L tetramer structure as the search model (PBD entry 1FAT) (Table 1). The crystal space group was found to be P1, and the asymmetric unit contained an *r*TBL-1 homo-tetramer composed of two antiparallel dimers (Figure 4A). Each monomer was composed of 15 antiparallel β-strands arranged in the classical β-sandwich or jellyroll fold observed for legume lectins such as in the case of their homologous PHA-E, PHA-L, SBA, PNA, ConA, and LOL, among others. The jellyroll fold consists of (1) a “back-sheet” with six β-strands, (2) a concave seven-stranded “front-sheet”, and (3) two “stranded-top” connecting sheets. Two divalent cations, calcium Ca^2+^ and manganese Mn^2+^, are attached to each protomer through coordination with Asp131, Glu129, Asp139, His144, Leu133, and Asn135 as well as two structural water molecules per ion. They are located at the top of the “front sheet” in the vicinity of the predicted carbohydrate binding site and are indispensable for lectin activity [37]. It is remarkable that the residues found to be involved coordination bonding of cations were identical to the ones previously described for its homologous PHA-L and very similar to the ones found in ConA, where the only mismatch corresponded to the substitution of Leu by Tyr [39].

Side-by-side, canonical dimers were formed by the antiparallel alignment of the “back” sheets of protomers A/B or C/D along β-strand 1 (amino acids 3–11; Figure 4AII). The tetramer was obtained from the back-to-back association of both dimers stabilized through interactions between the β-strands 10 (amino acids 186–194) of the corresponding chains (Figure 4AIII).

In accordance with the mass spectrometry data, we observed electron density that corresponds to the N-glycosylation site at Asn19. This permitted to build the proximal and the distal GlcNAc of the common core of N-glycans in all chains as well as six and four additional residues belonging to the antennas of chains A and D, respectively (Figure 4B). Those additional residues seem to be part of the structure Glc_3_Man_9_GlcNAc_2,_ which is the precursor of all *N*-glycans in eukaryotes. No evidence of hyperglycosylation or additional manα1-3 outer chains was identified. Considering the molecular weight obtained for the complete *N*-glycan of rTBL-1 during its characterization (~2.48 kDa) and the estimated molecular weight of the *N*-glycan identified by X-ray diffraction on chain A (~1.23 kDa), we assumed that there should be another ~7 residues on the antennas of rTBL-1. These could be a mixture of mannose and glucose which is isobaric to resemble the general structure of the *N*-glycan precursor. No structural evidence was found to suggest that the binding pockets could be affected by the presence and/or size of this posttranslational modification. However, the *N*-glycan seems to play a role in the folding of the protein and, in particular, in the stabilization of the dimer, since it favors the interfaces between the A/B and C/D chains (Figure 4AIV).

### 3.5. rTBL-1 Binding Properties

**Figure 5:**
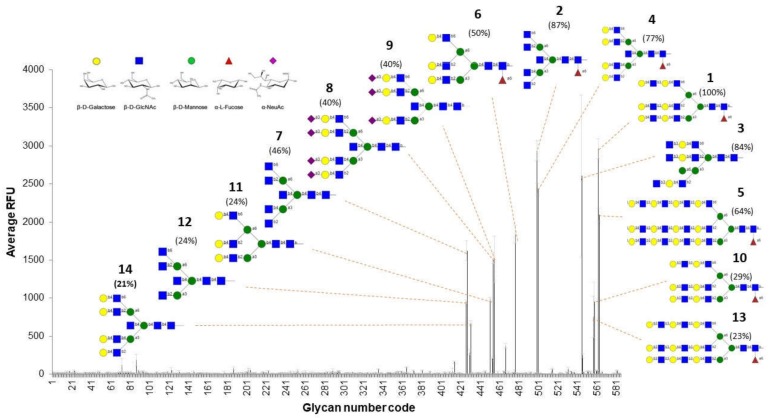
Glycan array results for rTBL-1 interaction with biologically relevant oligosaccharides: The bars show the average of the relative fluorescent units (RFUs) for each glycan of the matrix at 50 µg/mL of rTBL1-FITC. Oligosaccharide structures of the top binders are shown attached to their corresponding signal. The numbers that accompany each structure indicate the ascending order of binders, starting with one as the highest RFU% (percentage of relative fluorescent units with respect to the strongest binder) for each glycan, and are indicated in parenthesis. The order of the binders and the RFU% were estimated considering the results obtained at 5 and 50 µg/mL of rTBL1-FITC.

The glycan array was done by labeling the rTBL-1 with fluorescein isothiocyanate (FITC), followed by analysis at 5 and 50 μg/mL on the mammalian array “version 5.4” of the Consortium for Functional Glycomics. From the 585 glycans printed on the chip, only 14 were identified as binders (Figure 5). In all cases, rTBL-1 recognized β1-6 branched N-glycans independently of their size. Twelve of the binders contained a galactose residue linked to this branch; however, it does not seem to be a strict requirement for affinity, since glycans 2, 7, and 12 do not present this residue but were also recognized. This information contrasts with previous reports for other leucoagglutinins, as in the case of PHA:L from *P. vulgaris*, of which the minimal recognition unit for high affinity is the pentasaccharide Galβ1-4GlcNAcβ1-2[Galb1–4GlcNAcb1–6]Manα- and where galactose seems to influence the complex behavior [36,37].

*r*TBL-1 binding does not seem to be affected when the β1-6 branch is elongated with additional units of lactosamine (glycans 1, 5, 10, and 13) or α2-3 sialic acid (glycans 8 and 9). However, α1-6 fucosylated cores positively influence recognition. Binders 4 and 14 represent a clear example of this behavior, given that both have an identical structure—although binder 4 is a fucosylated core. The absence of this residue in binder 14 decreases the fluorescent signal of rTBL-1 by 56% with respect to its non-fucosylated counterpart. This same phenomenon can be observed for the glycan pairs 2/7 (41% decrease) and 6/11 (26% decrease).

The thermodynamics of binding with *N*-acetylglucosamine and other mono- and disaccharides, such as fucose, galactose, mannose, lactose, and sucrose, was approached by ITC; however, no binding was detected for any of the analyzed ligands. This, together with the glycan array matrix, shows that rTBL-1 binding is highly specific and requires complex *N*-glycans to display affinity.

These results are of the utmost importance, considering that β1-6 branched *N*-glycans have been described as one of the most common tumor-related glycosylation events [4,5,16]. The possible mechanisms by which increased β1-6 branched *N*-antennas enhance cancer progression include the lattice formation via galectin binding to LacNAc, leading to prolonged growth-factor signaling by EGFR [7]. Therefore, we posit that the cytotoxic effect that TBLF differentially displays between healthy and malignant cells could be related to the recognition of β1-6 branched *N*-antennas on EGFR. This would disrupt its function, either through internalization/degradation or through the impediment of ligand binding that transduces intracellular proliferation signals [4,5,16].

## 4. Conclusions

A single copy insertion of a TBL-1 coding sequence into the *P. pastoris* genome leads to the high-performance production of *r*TBL-1 lectin (316 mg/mL culture). The possibility of increasing yields via the insertion of multiple gene cassettes will be addressed. Modifications derived from the heterologous expression of *r*TBL-1 did not affect its folding or biological behavior. *r*TBL-1 is glycosylated, which does not interfere with binding to other glycans but does contribute to the structural maintenance of the tetramer, as noted in its resolved crystal structure. The absence or the elongation of the glycosylation, if produced in other heterologous expression systems, could affect the overall folding and functionality of the protein. *r*TBL-1 recognizes β1-6 branched *N*-glycans, which are overrepresented in several types of cancer, including colon cancer. The fucosylated core enhances *r*TBL-1–ligand affinity by an unknown mechanism.

We found an efficient system for the production of a recombinant lectin with anticancer potential in adequate yield to proceed with in vitro cytotoxicity assays on cancer cells and to perform in vivo tests. Currently, we are working on the co-crystallization of the *r*TBL-1 with ligands to obtain structural insights into its specific recognition by cancer-type glycans, and we are evaluating the effect of punctual mutations on its cytotoxic activity. The study of its interactions with membrane molecules with β1-6 branched N-antennas (like EGFR) is also being addressed.

## Figures and Tables

**Table 1:** Data collection and refinement statistics.

Data Collection					
Beamline		PX1, SOLEIL			
Wavelength (Å)		0.97918			
Space group		P1			
a, b, c (Å)		57.57, 64.47, 67.59			
α, β, γ (°)		96.35, 101.95, 97.27			
No. of monomers in asymmetric unit		4			
Resolution (Å)		32.73–1.9 (1.94–1.9)			
Total no. of reflections		451,693 (25,605)			
No. of unique reflections		73,400 (4491)			
Completeness (%)		99.7 (98.8)			
Multiplicity		6.2 (5.7)			
Mean I/σ (I)		11 (2.8)			
R*merge*		0.073 (0.529)			
R*meas*		0.088 (0.649)			
CC1/2		0.997 (0.902)			
Wilson B factor (A°^2^)		23.8			
**Refinement**					
Resolution (Å)	32.73–1.90				
No. of reflections	73,390				
No. of reflections in test set	3643				
R_work_/R_free_	0.169/0.213				
Rmsd bonds (Å)	0.014				
Rmsd angles (°)	1.846				
Rmsd Chiral (Å^3^)	0.091				
**No. Atoms/Bfac (Å^2^)**	**Chain A**		**Chain B**	**Chain C**	**Chain D**
Protein	1805/30.1		1779/30.7	1805/30.7	1747/31.7
Glycan	94/40.7		28/51.8	28/58.0	72/47.9
Metal ions	2/22.7		2/23.0	2/22.4	2/23.0
Water	129/37.1		131/36.5	136/37.0	124/36.5
Ramachandran Favored (%) Allowed (%) Outliers (%)	97 3 0				
PDB Code	6TT9				

Note: Values in parentheses are for the outer shell. PDB: Protein data bank.
